# The Genome of Banana Leaf Blight Pathogen *Fusarium sacchari* str. FS66 Harbors Widespread Gene Transfer From *Fusarium oxysporum*

**DOI:** 10.3389/fpls.2021.629859

**Published:** 2021-02-04

**Authors:** Yiping Cui, Bo Wu, Aitian Peng, Xiaobing Song, Xia Chen

**Affiliations:** ^1^Guangdong Provincial Key Laboratory of High Technology for Plant Protection, Plant Protection Research Institute, Guangdong Academy of Agricultural Sciences, Guangzhou, China; ^2^School of Computing, Clemson University, Clemson, SC, United States

**Keywords:** banana leaf blight, *Fusarium sacchari*, *de novo* assembly, comparative genomics, gene transfer

## Abstract

*Fusarium* species have been identified as pathogens causing many different plant diseases, and here we report an emerging banana leaf blight (BLB) caused by *F. sacchari* (*Fs*) discovered in Guangdong, China. From the symptomatic tissues collected in the field, a fungal isolate was obtained, which induced similar symptoms on healthy banana seedlings after inoculation. Koch’s postulates were fulfilled after the re-isolation of the pathogen. Phylogenetic analysis on two gene segments and the whole genome sequence identified the pathogen belonging to *Fs* and named as *Fs* str. FS66. A 45.74 Mb genome of FS66 was acquired through *de novo* assembly using long-read sequencing data, and its contig N50 (1.97 Mb) is more than 10-fold larger than the previously available genome in the species. Based on transcriptome sequencing and *ab initio* gene annotation, a total of 14,486 protein-encoding genes and 418 non-coding RNAs were predicted. A total of 48 metabolite biosynthetic gene clusters including the fusaric acid biosynthesis gene cluster were predicted *in silico* in the FS66 genome. Comparison between FS66 and other 11 Fusarium genomes identified tens to hundreds of genes specifically gained and lost in FS66, including some previously correlated with *Fusarium* pathogenicity. The FS66 genome also harbors widespread gene transfer on the core chromosomes putatively from *F. oxysporum* species complex (FOSC), including 30 involved in *Fusarium* pathogenicity/virulence. This study not only reports the BLB caused by *Fs*, but also provides important information and clues for further understanding of the genome evolution among pathogenic *Fusarium* species.

## Introduction

Bananas (*Musa* spp.) are one of the most important crops in the world that has been widely planted as staple food or fruit in many tropical and subtropical regions. China is one of the largest banana production countries, and most banana cultivars planted in China belong to the Cavendish subgroup of the AAA banana cultivar group (*Musa acuminata*). In recent years, the production of banana in China has been affected by several diseases, especially the catastrophic Panama disease (or Fusarium wilt) caused by *Fusarium oxysporum* f. sp. *cubense* (*Foc*) tropical race 4 (Ploetz, 2006; Dong et al., 2013), and new diseases have been keeping emerging (Fan et al., 2016; Du et al., 2017; Zhou et al., 2017). Immediate report of new diseases and understanding of their characteristics are essential for controlling their damage to the banana industry.

The genus *Fusarium* includes more than 20 monophyletic species complexes and several monotypic lineages involving many destructive plant pathogens (O’Donnell et al., 2015). In this study, the pathogen *Fusarium sacchari* (*Fs*) of the reported banana leaf blight (BLB) disease belongs to the *Fusarium fujikuroi* species complex (FFSC) which includes approximately 50 species (O’Donnell et al., 2015). FFSC is phylogenetically close to *Fusarium oxysporum* species complex (FOSC) which includes *Foc* (Maryani et al., 2019). *Fs* could cause growth disease on sugarcane (Bao et al., 2020; Yao et al., 2020) and oil palm (Suwandi et al., 2018), and postharvest fruit rot on lady finger banana (Riolo et al., 2020). Several other studies suggested that different *Fs* strains could harbor distinct pathogenicity toward bananas. An *Fs* isolate was isolated from banana fruit in 2013, but its pathogenicity was untested (Zeng et al., 2013). In another study, *Fs* and 10 other species were isolated from rot banana fruit, and only *Fs* isolates could not induce fruit rot after inoculation on healthy fruit (Abd Murad et al., 2017). Two FFSC isolates, *F. verticillioides* str. M108 and *Fs* str. M7, were isolated from pseudo-stem and leaves of *Musa* spp. ABB plants with Panama disease in Southern Mexico, respectively, but no evidence robustly supported they could cause symptoms through root inoculation (Maldonado-Bonilla et al., 2019). Overall, to our knowledge, *Fs* has not been related to any banana growth disease yet. Except for *Foc* and *Fs*, at least seven other *Fusarium* species have been linked to distinct banana diseases (Jones, 1997; Du et al., 2017). The large number of pathogenic *Fusarium* species and frequently identified new pathogenic strains toward bananas suggest that many *Fusarium* species have the potential to develop pathogenicity toward bananas.

Gene transfer through horizontal gene transfer (HGT) or introgression might have contributed to the development of new pathogenicity in the *Fusarium* genus. In recent years, more and more knowledge has been achieved on the impact of HGT on eukaryotic evolution, especially for unicellular organisms (Fitzpatrick, 2012), which refers to the non-sexual transmission of genetic material between organisms in nature (Keeling and Palmer, 2008). HGT could confer nutrition-related fitness (Milner et al., 2019) and modify the pathogenicity and host range (Alexander et al., 2016; Zhang et al., 2019) of fungi. In *Fusarium*, HGT of genetic materials has been observed in multiple species from divergent organisms (Ma et al., 2013; Sieber et al., 2014; Gao et al., 2019; Kim et al., 2020) and between close relatives within the genus (Liu et al., 2019; Bredeweg and Baker, 2020). Because no sexual cycle has been observed in FOSC, the homologous recombination events were all considered as HGT events in the group (Liu et al., 2019), but the possibility of introgression through rare inter-population or inter-species hybridization followed by multiple backcrossings could not be completely excluded. In FOSC, HGT of effector genes on the lineage-specific accessory or chromosomes has been related to pathogenicity gain and host range alteration (Ma et al., 2010; Laurence et al., 2015; van Dam et al., 2017; Czislowski et al., 2018). Recently, HGT has also been observed on multiple genes located on the core chromosomes within FOSC and between FOSC and FFSC (Liu et al., 2019; Bredeweg and Baker, 2020), suggesting that HGT in the genus could be more widespread than previously expected. HGT and introgression occur through different mechanisms and both cause gene transfer (Schmickl et al., 2017), and HGT could occur among more divergent organisms. When happening between phylogenetically close populations or species, the outcome of HGT and introgression could converge and be indistinguishable. Introgression was even considered as a path of HGT in some literature (Choudhuri, 2014; Bredeweg and Baker, 2020), which is controversial since it conflicts with the definition of HGT. In FFSC, teleomorphs have been observed in only about one-fifth of the species in the group including *Fs* (Leslie et al., 2005; Montoya-Martínez et al., 2019), suggesting that introgression could also have played a role in gene transfer from other species in *Fs* genomes.

In this study, we reported the BLB disease caused by *Fs* str. FS66 for the first time. The pathogen of the disease was confirmed by fulfilling Koch’s postulates. We acquired a high-quality genome assembly and the whole-genome gene annotation for *Fs*. Through comparative genomics and phylogenetic analysis, we also revealed the lineage-specific gene gain and loss and gene transfer at the whole-genome scale in the FS66 genome.

## Materials and Methods

### Plant Materials and Pathogen Isolation

Banana leaves showing leaf blight symptoms were collected from three different ‘Baxi’ banana plants in a banana orchard (113°58′ E, 23°34′ N) located in Longmen, Guangdong, in July 2016. Pieces (0.5 × 0.5 cm) were cut from the margin of the infected lesions, surface sterilized with 70% ethanol for 30 s, followed by 0.3% NaClO solution for 1–2 min, and then rinsed three times in sterile water. Three to five pieces from each original plant were placed on potato dextrose agar (PDA) medium and incubated in darkness at 25°C. Single spore cultures were obtained from each growing colony and subjected to morphological characterization.

### Inoculation of Candidate Pathogen on Banana Seedlings

Only one type of fungal colony was obtained from the last step. One isolate from each plant was subjected to a pathogenicity test. Two different inoculation methods were used to inoculate the isolates on 1-month ‘Baxi’ banana seedling leaves. In the first method, we dipped the leaves of three replicate seedlings in conidial suspensions (1.0 × 10^6^ CFU/ml) of each isolate for 10 s, and another three seedlings were dipped in distilled water as a control. In the second method, five puncture wounds were made on both sides of the leaves of banana seedlings using sterilized needles. On three replicate seedlings for each isolate, one side of each wounded leaf was inoculated with three conidial suspensions (1.0 × 10^6^ CFU/ml) drops (5 μl) of the isolate while the other half were treated with distilled water as a control. The plants from different treatment groups were grown in separate growth chambers at 25°C with a 16 h photoperiod each day. After leaf blight symptoms were observed on the inoculated leaves, the fungus was re-isolated using the same method described in the previous section. The isolates from different plants were later confirmed to be identical and named FS66, which was also inoculated onto five pseudo-stems of 1-month old ‘Baxi’ banana seedling using both the methods applied on the leaves. The same number of negative controls were inoculated with water. To test if the isolate could invade through banana roots, we applied root inoculation on five 1-month old ‘Baxi’ banana seedlings as described in the study of Araújo et al. (2017). A pathogenic *Foc* tropical race 4 isolate was inoculated as the positive control, and distilled sterile water was used as the negative control on an equal amount of seedlings. The plants were kept in the greenhouse, and the symptom development was recorded over 2 months.

### Extraction of DNA and RNA

DNA mixture of the host plant and the pathogen was extracted from symptomatic banana leaf tissues was carried out using the modified CTAB method (Gawel and Jarret, 1991). Total genomic DNA was extracted from three single conidium cultures from different plants grown on PDA using the AxyPrep Multisource Genomic Miniprep DNA kit (Axygen, New York, NY, United States) following the instruction. RNA for transcriptome sequencing was extracted from one single conidium fungal culture using the AxyPrep Multisource Genomic Miniprep RNA kit (Axygen, New York, NY, United States). The quality of the DNA and RNA were checked using both Nanodrop 2000c Spectrophotometers (Thermo Fisher Scientific, Waltham, MA, United States) and agarose gel electrophoresis.

### Detection of Putatively Existing *Foc* in Symptomatic Tissues

We applied the *Foc* detection method described by Li et al. (2012) on both DNA extracted from the symptomatic leaf tissues and that from the fungal culture, except that primers in the multiplex PCR for amplifying the race 1 and race 4 were run in separate tubes. The *Foc* race 1 specific primers are W1805F: 5′-GTTGAGTCTCGATAAACAGCAAT-3′, and W1805R: 5′-GACGAGGGGAGATATGGTC-3′. The *Foc* race 4 specific primers are W2987F: 5′-GCCGATGTCTTCGTCAGGTA-3′ and W2987R: 5′-CTGAGACTCGTGCTGCATGA-3′. The amplicons were subjected to agarose gel electrophoresis, and PCR amplicons using DNA of our previously obtained *Foc* race 1 and race 4 isolates FOC1 and FOC4 as templates were added as the positive control.

### Identification of the Pathogen by Molecular Phylogeny

To determine the taxon of the pathogen, we sequenced a segment of the internal transcribed spacer (ITS) and partial coding sequence of the second-largest subunit of RNA polymerase II (*RPB2*) of three isolates from three different plants. The primers used for ITS amplification were ITS1 and ITS4 (White et al., 1990), and RPB2-7cR and RPB2-5F (Reeb et al., 2004) were applied in amplification of the *RPB2* segment. Each amplification reaction included 25 μl of 2 × EasyTaq PCR SuperMix (TransGen Biotech, China), 2 μl (10 mM) of each primer, and 17 μl of double-distilled water in a final volume of 50 μl. The amplification program was set as follows: an initial denaturation step at 94°C for 5 min, followed by 36 cycles consisting of 30 s at 94°C, 1 min at 54°C, and 5 min at 72°C, and a final elongation step of 10 min at 72°C. PCR reactions were performed on a BIO-RAD T100^TM^ PCR machine (Bio-Rad Laboratories, Hercules, CA, United States). Sanger sequencing of the amplicons was carried out by Sangon Biotech (Shanghai, China).

We searched the sequences of both the segments against the NCBI nt (non-redundant nucleotide) database using BLASTN algorithm (Boratyn et al., 2013), and sequences of a few isolates from *Fs* and several close *Fusarium* species were downloaded for phylogenetic analysis. Multiple sequence alignment was carried out using Clustal X v2.1 (Larkin et al., 2007), and phylogenetic trees were constructed using both Neighbor-joining (NJ) and Maximum likelihood (ML) methods implemented with 1000 rounds of bootstrap tests in MEGA X v10.1 (Kumar et al., 2018).

### Optimal Temperature and pH for the Pathogen Growth on PDA

We carried out three replicates for each culture condition in this section. To find the optimal temperature for the growth of FS66, we inoculated it on PDA (pH = 7.0) plates and kept them in incubators set at 5, 10, 20, 25, 30, 35, and 40°C under darkness. To study its tolerance to different pH values, we inoculated it on PDA mediums adjusted to pH 2.0, 3.0, 4.0, 5.0, 6.0, 7.0, 8.0, 9.0 with NaOH and HCl solutions, which were incubated in darkness at 25°C. The diameters of the colonies were measured and recorded for a week after inoculation.

### Whole-Genome Sequencing and *de novo* Assembly

The whole genome of FS66 was sequenced using both PacBio sequencing and next-generation sequencing (NGS) by NovoGene (Beijing, China). PacBio sequencing libraries were constructed following the 20 kb SMRTbell^TM^ (Pacific Biosciences, CA, United States) protocol and subjected to sequencing on the PacBio Sequel System (Pacific biosciences, CA, United States). We acquired 1,682,281 PacBio sequencing reads totaling 13.39 Gb, with an average length of 7,959 bp and an N50 of 10,432 bp. For NGS, a library with on average 350 bp insertion size was constructed, and 2 × 150 bp pair-end sequencing was implemented on an Illumina HiSeq 4000 instrument (Illumina, CA, United States). We acquired a total of 2.18 Gb clean reads by NGS.

*De novo* assembly using Pacbio reads was carried out with Miniasm v0.3 (Li, 2016). Polishing of the assembly was carried out using Racon v1.4.3 (Vaser et al., 2017) and Pilon v1.23 (Walker et al., 2014) with Pacbio reads and NGS sequencing reads respectively. The mitochondrial genome was manually corrected by integrating contigs and removing redundant regions caused by the circular topology at the ends. The assembly parameters, including N50, L50, et al., were evaluated using QUAST v5.0.2 (Gurevich et al., 2013).

### Transcriptome Sequencing and Assembly

The RNA extracted from the fungal culture was subjected to RNA-seq by NovoGene (Beijing, China). mRNAs were enriched with oligo(dT) before the 350 bp insertion-size pair-end RNA-seq library was constructed. High-throughput sequencing was carried out on an Illumina HiSeq 4000 instrument and a total of 7.35 Gb pair-end reads (2 × 150 bp) were acquired. The RNA-seq data were mapped to the FS66 assembly by HISAT2 v2.2.1 (Kim et al., 2019) and assembled using both StringTie v2.1.4 (Pertea et al., 2015) and Class2 (Song et al., 2016). Then we picked up the high-quality assembled transcripts with intact coding regions from transcriptome sequencing using Mikado v1.5 (Venturini et al., 2018).

### Gene Structure Annotation

Mitochondrial and nuclear genomes were annotated separately for FS66. We predicted the repetitive elements in the genome with the Extensive *de novo* TE Annotator (EDTA) pipeline (Ou et al., 2019) and soft masked the assembly before *ab initio* gene structure annotation. Nuclear protein-encoding genes were predicted using both *ab initio* prediction and evidence-based inference methods. First, the high-quality transcripts assembled in the last step were mapped to FS66 via minimap2 v2.17 (Li, 2018) in long mRNA read mode. Based on the transcript assembly and *Fusarium* protein sequences downloaded from NCBI database, Augustus v3.2.3 (Stanke et al., 2006) was trained in *ab initio* gene prediction for FS66 following the protocol of Hoff and Stanke (2019). Homology based annotation was carried out by GeMoMa v1.7.1 (Keilwagen et al., 2019) using *F. fujikuroi* genome (GCA_900079805.1) as reference. We merged the annotation files from RNA-seq data assembly, homology-based annotation, and *ab initio* prediction, which were further subjected to redundancy removal using GffRead v0.12.3 and GffCompare v0.12.1 (Pertea and Pertea, 2020). Coding regions were inferred from all transcripts using TransDecoder v5.5.0^1^. The completeness of the gene structure annotation was assessed with BUSCO v4.0.5 (Simão et al., 2015). The function of the whole-genome genes was annotated by searching against the InterPro database (Hunter et al., 2009) and KEGG database (Kanehisa et al., 2017) through BLAST. GO annotation of all protein-encoding genes was carried out using BLAST2GO v5.2.5 (Götz et al., 2008).

We applied both RNAmmer v1.2 (Lagesen et al., 2007) and Barrnap v0.9^2^ in predicting 5S, 5.8S, 18S, and 28S rRNAs in the genome. The tRNAs were predicted using tRNAscan-SE v2.06 (Chan and Lowe, 2019). Other non-coding RNAs including small nuclear RNA (snRNA), small nucleolar RNAs (snoRNAs), spliceosome RNAs, et al. were annotated via homology analysis on both sequence and secondary structure by Rfam 14.3 (Kalvari et al., 2018) and Infernal v1.1.2 (Nawrocki and Eddy, 2013). Genes encoding proteins and non-coding RNAs in the mitochondria genome were predicted using MITOS2 web server^3^ (accessed on 10/03/2020) with the reference set as ‘RefSeq 63 Fungi’ and genetic code as ‘4 mold’ (Bernt et al., 2013).

All *F. fujikuroi* mating-type proteins (Martin et al., 2011) including MAT 1-1 (G3G2C1, G3G2C2, G3G2C3, O93924, O93924, O93925, Q9C461, Q9URK7) and MAT 1-2 (G3G2C4, G3G2C5, O93926, O94158) types were downloaded from the UniProt database. A local blast database was created using all the annotated proteins in FS66 genome, and all the MAT proteins were used as query sequences to search for their orthologs in FS66.

All pathogenicity or virulence-related *Fusarium* genes in the Pathogen Host Interactions (PHI) database (Urban et al., 2017) were searched against the acquired orthogroups using MMseqs2 v12.113e3 (Steinegger and Söding, 2017), and the best hits were considered as the orthologous groups to the query genes.

### Identification of Gene Orthogroups From Multiple Fusarium Genomes and Species Tree Construction

The genome sequences of several *Fusarium* species (Ma et al., 2010; Wiemann et al., 2013; Niehaus et al., 2016, 2017; King et al., 2018; Asai et al., 2019; Warmington et al., 2019; Wang et al., 2020) close to FS66 were downloaded from GenBank assembly database (Supplementary Table 1). We assessed the completeness of their gene structure annotations with BUSCO v4.0.5. We required the genomes to have no less than 98% tested genes classified as complete to include them in the further analysis except for the *Fs* str. NRRL 66326 (GCA_013759005.1), which was an isolate derived from the mating of two isolates collected from sugarcanes in Taiwan and the only available *Fs* genome in the public database. The genome of *F. venenatum* str. A3/5 (GCF_900007375.1) was selected as an outgroup. No gene structure annotation was available for *Fs* str. NRRL 66326, so we predicted the protein-encoding genes in the genome using GeMoMa v1.7.1 with FS66 as reference. Whole-genome alignment between FS66 and NRRL 66326 or FO4287 (GCA_000149955.2) and genetic variation identification were carried out using Mummer v4.0.0.beta5 (Marçais et al., 2018).

Whole-genome protein sequences were obtained for the 12 analyzed genomes either through downloading from NCBI GenBank database or output by GffRead v0.12.3. For genes annotated with more than one alternatively spliced transcripts, only proteins encoded by the main transcripts were used. To identify the orthologous and paralogous (duplicated) relationship among the genes, the OrthoFinder v2.4.0 pipeline (Emms and Kelly, 2019) was then applied to all the proteins. The pipeline includes the following step: (1) the proteins were clustered into orthogroups based on normalized genetic distance. An orthogroup has been defined as homologous genes from extant species which were descended from a single gene in the last common ancestor of the species; (2) multiple alignments and maximum likelihood tree construction were carried out for each orthogroup; (3) A species tree was constructed by STAG v1.0.0 (Emms and Kelly, 2018) based on all the individual gene trees. The supporting rate of each bipartition was calculated as the proportion of gene trees with the bipartition, which has been supposed to be a more stringent measure than standard bootstrap support (Emms and Kelly, 2018); (4) Gene duplication events were inferred based on the species tree and gene trees.

### Gene Gain and Loss

We obtained the number of gene copies in each orthogroup for each genome from the results of the OrthoFinder pipeline. A *t*-test was carried out to check if there was a significant (*p* < 0.01) difference in the gene copy number in each orthogroup between FSCC and FOSC genomes. Gene gain and loss in FS66 compared with other FSCC genomes were analyzed using the following approach. In each orthogroup, the mean value(*m*) and the standard deviation(*s*) of gene number in the six FSCC genomes (not including FS66 and NRRL 66326) were calculated. If the number of genes of FS66 in an orthogroup is smaller than (*m* – 2 × *s*) or larger than (*m* + 2 × *s*), gene loss or gene gain was inferred in FS66, respectively.

### Gene Cluster Prediction and Comparison

Putative gene clusters involved in secondary metabolites biosynthesis were predicted using antiSmash v5.1.2 (Blin et al., 2019) locally, and the borders of the gene clusters were inferred using the implemented cassis algorithm. The gene clusters from the 12 genomes were merged and formatted into a MultiGeneBlast database, and each predicted gene cluster was searched against the database using MultiGeneBlast v1.1.14 as done by Hoogendoorn et al. (2018).

### Inference of Genomic Regions Transferred From Other Species

The FS66 assembly was split into 1 kbp sliding windows with 500 bp overlap across the genome using Bedtools v2.29.2 (Quinlan, 2014). The genomic segments in these windows were aligned using BLASTN to the previously compared 11 Fusarium genomes and another three outgroup *Fusarium* genomes, *F. tricinctum* (GCA_900382705.2) (Ponts et al., 2018), *F. graminearum* (GCA_900044135.1) (King et al., 2015), and *F. avenaceum* (GCA_000769295.1) (Lysøe et al., 2014). These genomes except for FS66 were classified into FFSC (seven genomes), FOSC (three genomes), and the outgroup (four genomes). When the best BLASTN hit segment in a genome is longer than 500 bp and shares >50% nucleotide similarity with the query FS66 segment, it was identified as an ortholog of the query. Each query segment from FS66 and all its orthologs found in the 14 genomes were output in an individual fasta file. When at least one ortholog of a segment was available in each of the FFSC, FOSC, phylogenetic incongruence test was carried out to check if there was gene transfer from other groups. Because the FFSC species were phylogenetically close and incomplete lineage sorting could also result in many incongruent gene trees (Maddison and Knowles, 2006), only gene transfer from FOSC was inferred by this test. To reduce the impact of gene transfer in the other genomes on our analysis, the test was carried out on all the possible ortholog combinations among FS66 and the three groups. The test includes the following steps: (1) one ortholog was randomly selected from each of the three groups and output together with the corresponding FS66 segment into a FASTA file, and all the available combinations were output in separate files for each FS66 window; (2) all the FASTA files were subjected to multiple alignments with MUSCLE v3.8.1551 (Edgar, 2004) and ML tree search using RAXML v8.2.12 (Stamatakis, 2014), and the best tree in 100 iterations of ML tree search was acquired for each FASTA file; (3) the DendroPy v4.4.0 (Sukumaran and Holder, 2010) python phylogenetic package was applied to check if the best ML tree inferred from each FASTA file supported local gene transfer from FOSC in FS66; (4) FS66 genome windows with more than 50% FASTA files supporting gene transfer were considered as genomic regions putatively transferred from FOSC. All the circular graphs in this study were drawn using Circos v0.69-5 (Krzywinski et al., 2009).

## Results

### Banana Leaf Blight Disease Observed in Guangdong, China

**FIGURE 1:**
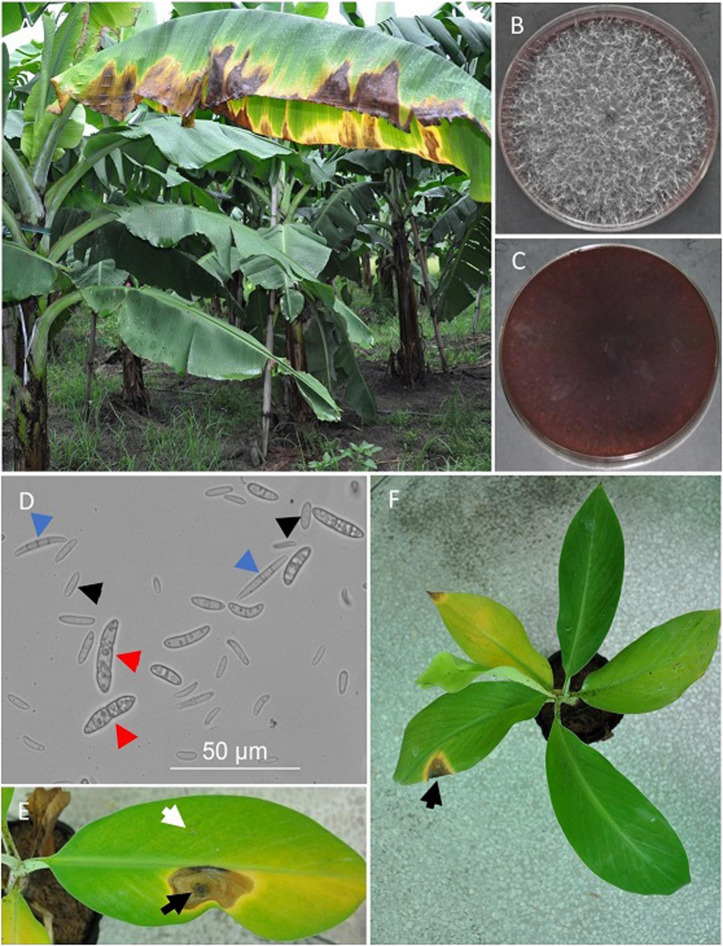
Banana leaf blight disease and pathogen isolation. **(A)** Symptoms of the BLB disease in the field. **(B,C)** The appearances of the fungal colony at 7 days after single-spore inoculation on PDA plates from the front **(B)** and the back **(C)**. **(D)** Morphology of the fungal conidia and spores under the optical microscope. Blue, black, and red arrows denote large conidia, small conidia, and ascospores, respectively. **(E)** Symptoms on leaves inoculated with both the candidate pathogen and sterile water after the puncture. The white and black arrows pointed to the area punctured with needles and inoculated with water and FS66, respectively. **(F)** Symptoms on a banana seedling treated with leaf non-wound inoculation. Pictures **(E,F)** were taken at 30 days after inoculation.

In July 2016, leaf blight was observed on about 20% ‘Baxi’ (*Musa* spp. AAA Cavendish) plants in a 2 ha banana plantation located in Longmen, Guangdong. Yellow to brown lesions expanded from the leaf blade edge to the inner area were observed on the symptomatic tree (Figure 1A). The symptoms were more often observed on the young leaves than older leaves. A few trees (∼5% of the infected trees) developed very severe symptoms and stopped growing months after the symptom first appeared. The disease was continuously observed in the following 3 years, and it was more severe and widespread between early May and late September when the amount of rainfall was relatively high within a year in the region. We dissected the roots and pseudo-stems of five diseased trees with the most severe symptoms, and no discoloration of vessels was observed, showing it was not a wilt disease.

### Isolation of a Fungal Pathogen of the Disease

The same type of reddish-brown colonies with abundant white wooly mycelia was obtained ∼2 days after the inoculation of the symptomatic tissues from three different trees on PDA (Figures 1B,C). The mycelia were straight, septate, and only a few branches were observed. There were two different types of conidia (Figure 1D): the large conidia were falcate, septal, hyaline, and 11.88 ∼ 63.5 μm × 2.5 ∼ 6.2 μm in size; the small conidia were elliptical, hyaline, and 5 ∼ 10.5 μm × 2.0 ∼ 4.8 μm in size. We also observed some ascospores, which were finger-shaped, containing at least one septum, and 11.5 ∼ 95 μm × 3.5 ∼ 7.5 μm in size (Figure 1D). As shown in Supplementary Figure 1, the optimal temperature and pH of the isolates were identified to be around 25°C and 7.0, respectively (Supplementary Figure 1).

Three isolates from different trees were inoculated on banana leaves and pseudo-stems through both non-wound and wound inoculations. Both types of tissues with puncture wounds started developing blight symptoms as early as 4 days after inoculation with the candidate pathogen, and the symptoms on leaves and pseudo-stems at 30 days and 45 days after inoculation were shown in Figure 1E and Supplementary Figure 2, respectively. No symptom was observed on any of the negative controls inoculated with water. When non-wound inoculation of the isolates was applied to the leaves, only two of the nine seedlings developed leaf blight symptoms (Figure 1F). None of the five pseudo-stems subjected to non-wound inoculation developed any symptom. Five banana seedlings were subjected to root inoculation of the candidate pathogen, and no symptom was observed on any of them, while all positive controls inoculated with a pathogenic *Foc* tropical race 4 isolate developed symptoms of the Panama disease as early as 15 days after inoculation.

### Identification of the Taxonomy of the Pathogen

Considering Panama disease had been detected in adjacent orchards, we suspected there could be *Foc* involved in the disease at the beginning and carried out PCR test using both *Foc* race 1 and race 4 specific primers. With either the DNA extracted from symptomatic plant tissues or the fungal isolates as templates, no *Foc* target amplicon was obtained using either *Foc* race 1 or race 4 specific primers. Instead, a >1000 bp amplicon was amplified by the race 1 primers, and no (symptomatic plant tissues DNA as templates) or only a very weak amplicon band (fungal culture DNA as templates) shorter than the target amplicon was observed for race 4 primers (Supplementary Figure 3).

**FIGURE 2:**
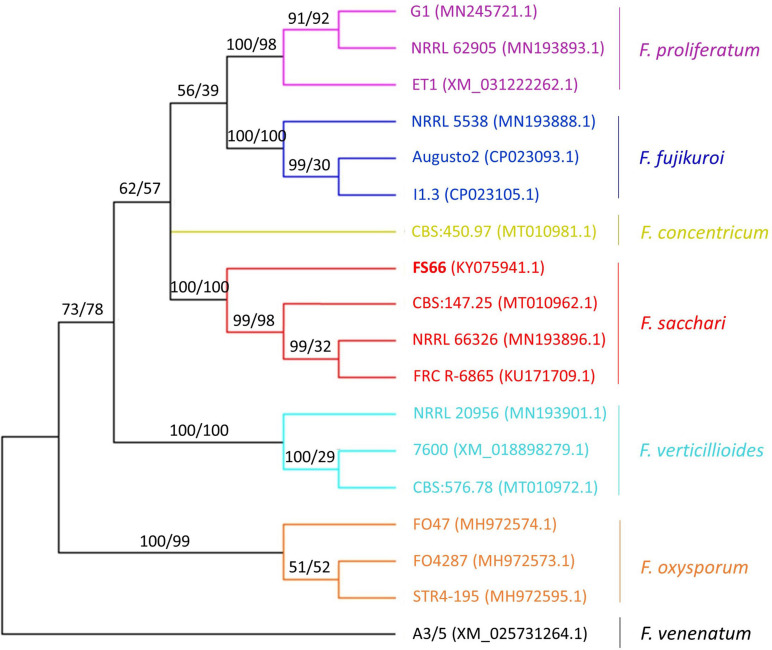
Phylogenetic tree based on the partial coding sequence of *RPB2*. The strain names and NCBI accession IDs (in brackets) are shown at the tips. The phylogenetic trees constructed by neighbor-joining (NJ) and maximum likelihood (MX) have the same topology, and the bootstrap support rates (percentage) from NJ and MX are shown before and after the slash next to the bipartition nodes, respectively. Branches corresponding to partitions reproduced in less than 50% bootstrap replicates have been collapsed.

We sequenced the internal transcribed spacer (ITS, KY075940.1) and segment the second-largest subunit of RNA polymerase II (*RPB2*, KY075941.1) segments of the three isolates from different trees. The results showed that the two segments shared 100% nucleotide similarity among them. Considering the three isolates had nearly the same colony phenotypes and pathogenicity, they were supposed to be the same fungal strain which we named FS66. Through searching in the NCBI nt database, we found that the ITS and *RPB2* segments of FS66 shared the highest nucleotide similarity (both 100%) with *Fs* isolates. Phylogenetic analysis based on ITS showed that FS66 should belong to FFSC, and *RPB2* supported that FS66 was clustered with the *Fs* strains (Figure 2). Based on the two segments and the whole-genome assembly in the following section, we concluded that FS66 belongs to *Fs*.

### *De novo* Assembly and Gene Structure Annotation of FS66 Genome

**FIGURE 3:**
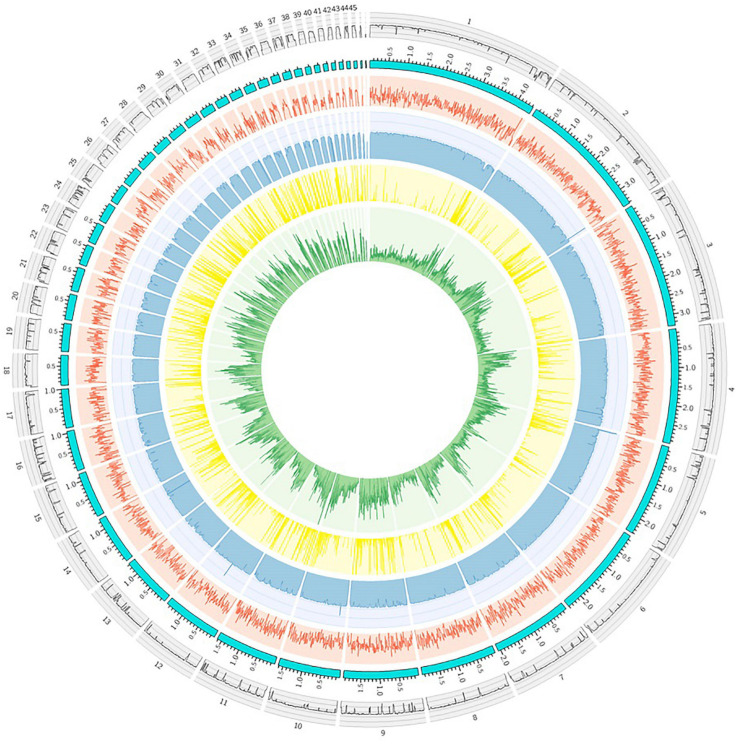
Distribution of genes, RNA-seq reads, and genetic variations across the FS66 genome. The data shown in this graph were calculated in 10 kbp continuous non-overlapping windows across the genome. From outer to inner: the GC content (GC%) panel (black); the ideogram (sky blue) of the 47 non-organelle contigs arranged by contig size; the gene count panel (red) showing the number of annotated genes; the RNA-seq panel (light blue) in which the log_2_ (read count mapped in each window) was shown; the unaligned region panel (yellow) which depicted the unaligned length between FS66 and *Fs* str. NRRL 66326 in each window; the SNP count panel showing the number of SNPs (including small indels) between FS66 and *Fs* str. NRRL 66326.

We sequenced *Fs* strain FS66 using both PacBio long-read sequencing (∼290×) and NGS (≥40×), based on which *de novo* assembly was carried out. The acquired genome consisted of 47 nuclear contigs and a complete 59,755 bp circular mitochondrial genome, totaling 45.74 Mb with an average GC content of 46.30% (Table 1 and Figure 3). The FS66 assembly has highcompleteness, and both high mapping rates of whole-genome sequencing reads (97.96%) and RNA-seq reads (93.61%) were achieved. The FS66 assembly is about 2.99 Mb larger than *Fs* str. NRRL 66326 (GCA_013759005.1) (Table 1), and its contig N50 size (1,965,716 bp) is more than 10-fold larger than that of NRRL 66326 (N50 = 187,816 bp). The largest contig of our FS66 assembly is 4,434,236 bp long, and a minimum of 8 and 16 contigs could cover >50% and >75% of the genome, respectively. The whole-genome alignment showed an overall nucleotide similarity of 96.74% between FS66 and NRRL 66326. Only 85.53% of the entire FS66 genome and 91.42% of the NRRL 66326 genome could be aligned with each other, and the single nucleotide variations (SNV) between the two genomes were distributed heterogeneously across the genome (Figure 3). The FS66 assembly was also aligned to the genome *F. oxysporum* f. sp. *lycopersici* 4287 (FO4287), and the four lineage-specific accessory chromosomes in FO4287 (Ma et al., 2010) were almost completely absent (99.7%) in FS66.

**TABLE 1:** Statistics on two *Fusarium sacharri* genome assemblies.

*Fs* strain name	FS66	NRRL 66326
GenBank ID	JADANP000000000	GCA_013759005.1
Number of contigs	48	479
Largest contig (bp)	4,434,236	997,752
Total length (bp)	45,739,938	42,740,848
N50 (bp)	1,965,716	187,816
N75 (bp)	1,048,338	96,836
L50	8	63
L75	16	142
GC (%)	46.3	49.03

We carried out gene structure annotation for FS66 based on transcriptome sequencing and *ab initio* gene prediction. A total of 14,486 protein-encoding genes were predicted in the genome (Figure 3). Assessment by BUSCO showed that 99.5% of tested Hypocreales core genes (4,494) were successfully annotated as complete BUSCOs in our annotation, which is as high as the best genome assemblies in several other *Fusarium* species. Besides protein-encoding genes, 418 non-coding RNAs, including 309 tRNAs, 67 rRNAs, and 42 other non-coding RNAs were predicted in the genome. The mitochondrial genome was annotated separately, in which 29 protein-encoding genes, 3 rRNAs, and 27 tRNAs were predicted. Gene functional annotation including Gene Ontology (GO), InterPro annotation, and KEGG pathway annotation were obtained for all the nuclear and mitochondrial protein-encoding genes.

### Gene Gain and Loss in FS66 Compared With Close *Fusarium* Species

**FIGURE 4:**
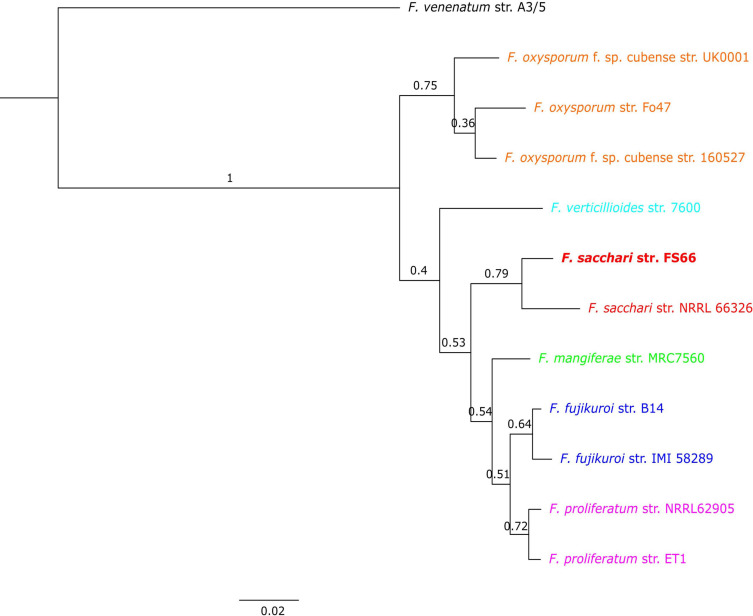
The best supported phylogenetic tree based on whole-genome proteins of FS66 and several adjacent *Fusarium* species. The species tree has been constructed based on the 9050 orthogroups, which include members from all the analyzed genomes. The lengths of the branches are proportional to genetic distance. The number on top of the branches denote the bipartition’s support rate at their right ends, which is the proportion of orthogroups supporting the bipartition. The number below each branch indicates the number of identified gene duplication events.

Orthologous gene groups (orthogroup) were inferred among FS66 and 11 other *Fusarium* genomes, including seven from FFSC, three from FOSC, and one from *F. venenatum* as outgroup (Supplementary Table 1). A total of 18,151 orthogroups were detected (Supplementary Table 2), 14,126 (77.8%) of which included genes from no less than six of the genomes (Supplementary Figure 4). There were 9050 orthogroups with all species present and 7725 of these consisted entirely of single-copy genes. A species tree for FS66 and six other Fusarium species was obtained based on the orthogroups, which confirmed that FS66 is the closest to *Fs* str. NRRL 66326 and belongs to FFSC (Figure 4).

**FIGURE 5:**
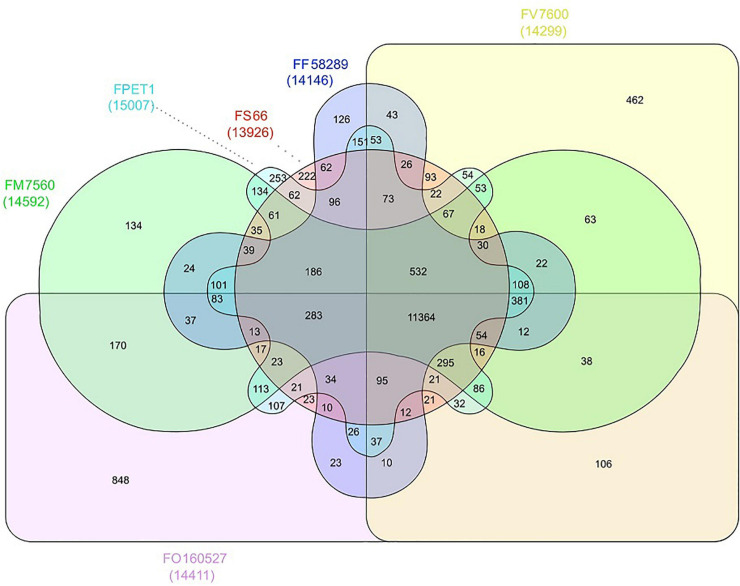
Orthogroup sharing relationships among six *Fusarium* genomes. The first two characters in the isolate names denote the species and the rest characters were the strain names listed in Supplementary Table 1. For some isolates, alphabet characters such as ‘NRRL’ were omitted for convenience. FM, *F. mangiferae*; FP, *F. proliferatum*; FF, *F. fujikuroi*; FV, *F. verticillioides*; FO, *F. oxysporum*.

FS66 has at least one gene in 13,866 orthogroups, and it generally shares more (on average 823.3) orthogroups with FFSC genomes than with FOSC genomes (Figure 5 and Supplementary Table 3). In 347 and 246 orthogroups, FFSC genomes have significantly (*p* < 0.01) fewer and more gene copies than the FOSC genomes, respectively. In comparison to other FFSC genomes, FS66 had gene copy gain in 539 orthogroups and gene copy loss in 778 orthogroups. Through phylogenetic analysis on all individual genes a total of 40 lineage-specific gene duplications were detected in FS66 (Figure 4). Four of the orthogroups with gene gain and nine with gene loss in FS66 have been correlated with phytopathogenicity in other *Fusarium* species in the Pathogen Host Interactions (PHI) database (Supplementary Table 4). The lost genes included three genes involved in the biosynthesis of fumonisins, FUM6, 8, and 21. No ortholog of mating-type MAT1-1 protein-encoding gene was found in FS66, and two MAT 1-2 type protein genes, *MAT 1-2-1* and *MAT 1-2-9*, were identified in the genome, indicating that FS66 belongs to the MAT1-2 mating type.

**FIGURE 6:**
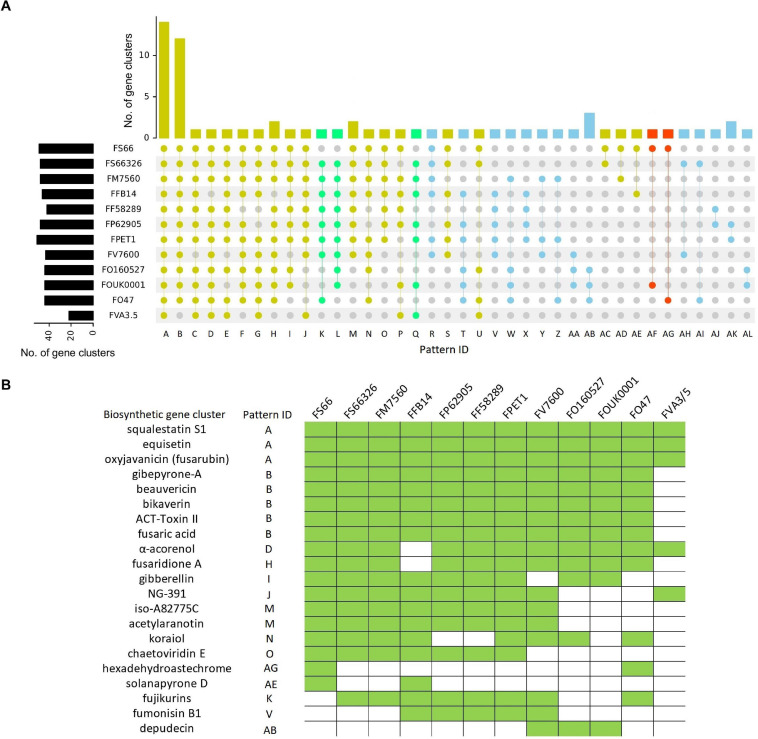
Secondary metabolite gene cluster distribution in 12 *Fusarium* genomes. The isolates were named in the same way as in Figure 5 except for FVA3/5 in which ‘FV’ denotes *F. venenatum*. **(A)** Distribution of gene cluster sharing patterns among the 12 genomes in a total of 67 gene clusters. In the bottom panel, gray dots indicate absence, while colored dots (yellow, blue, red, and green) show the presence of the gene clusters in the corresponding genomes. The patterns in green color denote specific gene cluster loss in FS66, red denotes gene clusters putatively transferred from FOSC in FS66, yellow patterns include gene clusters present in more than 50% genomes, and blue pattern gene clusters were present in less than 50% genomes. **(B)** Availability of previously annotated gene clusters in the genomes. Green rectangles indicate the presence of the biosynthetic gene clusters in the corresponding genomes.

All secondary metabolite biosynthesis gene clusters were predicted in the 12 *Fusarium* genomes. As a result, a total of 67 putative secondary metabolite biosynthesis gene clusters were detected, of which 48 were detected in the FS66 genome (Figure 6A and Supplementary Table 5). Among the 67 gene clusters, 21 are homologous to previously annotated gene clusters in the Minimum Information about a Biosynthetic Gene cluster (MIBiG) database (Figure 6B), including 18 existing in the FS66 genome. Three gene clusters present in *Fs* str. NRRL 66326 and at least one other FFSC genome were absent in FS66, including the annotated fujikurins biosynthesis gene clusters (BGC0001305). As shown in Figures 6A,B, at least three gene clusters were putatively transferred from FOSC or more divergent species to *Fs* (black arrow) or FS66 (red arrows), including the previously annotated hexadehydroastechrome biosynthetic gene cluster (BGC0000372).

### FS66 Genomic Regions Putatively Transferred From Other Species

Genomic regions putatively transferred from FOSC or other non-FFSC species in the FS66 were investigated. The whole-genome of FS66 was divided into 1 kbp windows and searched against the above 11 *Fusarium* genomes and 3 additional outgroup *Fusarium* genomes. Homologous regions of 92.3% (84,341/91,383) of the segments were detected in at least one genome. FS66 shared 65,399 to 78,302 homologous segments with the FFSC genomes, 64,732 to 64,996 segments with the FOSC genomes, and only 11,522 to 15,505 segments with the outgroup genomes. A total of 56 homologous segments (38 regions) involving 11 genes (three fully covered and eight partially covered) were only present in the outgroup.

**FIGURE 7:**
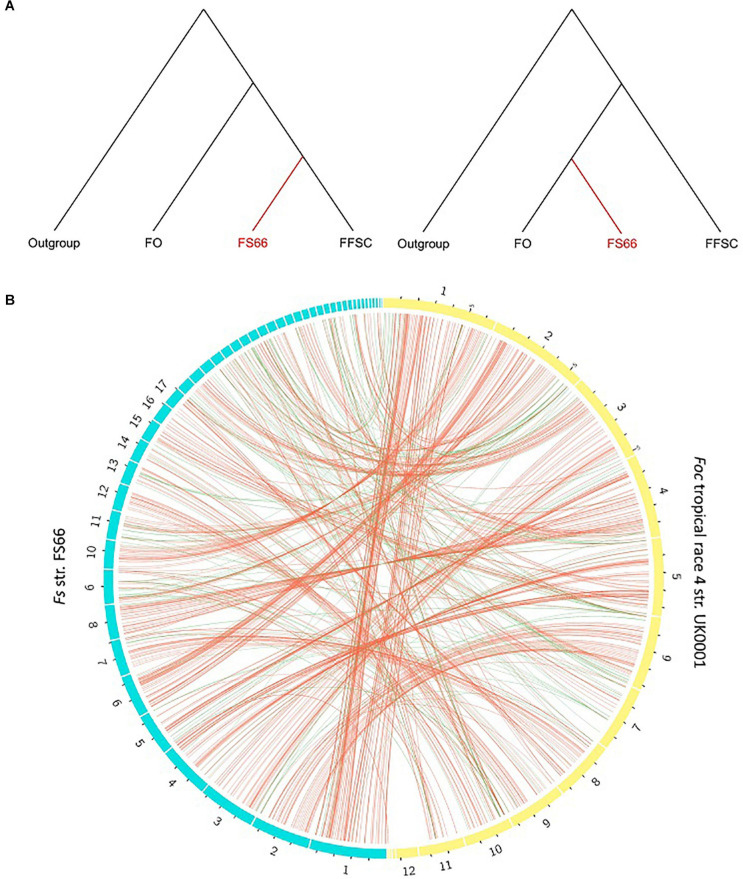
Putative genomic regions transferred from FOSC in the FS66 genome identified by phylogenetic incongruence analysis. **(A)** *S*pecies tree (left) and the incongruent molecular tree (right) suggesting gene transfer from FOSC in FS66. **(B)** Genomic regions putatively transferred from FOSC across the FS66 genome. Red links were inferred by phylogenetic incongruence analysis and green links were inferred based on that they were shared between FS66 and FOSC but not found in other tested FFSC genomes.

On 351 segments (221 regions), the homologs were only detected in the FOSC group but not in any of the other FFSC genomes or the four outgroup genomes, suggesting they could be derived from FOSC or its close relatives. In Figure 7B, 207 of the 351 segments have been shown as green links. On 21,827 FS66 genomic segments on which homologs were identified, genomic segments in FS66 putatively introduced from FOSC were further inferred based on phylogenetic incongruence between the species tree and the molecular trees (Figure 7A). Incongruence was detected on additional 555 segments (497 discontinuous regions), of which 546 have been shown as red links in Figure 7B. All these 960 segments which were putatively introduced from FOSC accounted for 814,500 bp in the genome, covering 27 genes entirely and 529 genes partially. Orthologs of 30 of the overlapped genes have been associated with pathogenicity and/or virulence in at least one *Fusarium* species (Supplementary Table 6), including two genes *FoSlt2* and *CHS2* reported to be related to the virulence of *F. oxysporum*. Considering only 23.9% (21827/91383) of genomic segments were subjected to the phylogenetic analysis due to lack of enough outgroup segments, there still could be many un-detected transferred regions in FS66.

## Discussion

In this study, we reported the BLB disease caused by *Fs* str. FS66 in the world for the first time, which is another disease on banana caused by a *Fusarium* pathogen. Previously, *Fs* strains were isolated from banana fruit and leaves in Asia, but they either had no (Abd Murad et al., 2017) or untested (Zeng et al., 2013) pathogenicity, suggesting the FS66 strain could have evolved the pathogenicity toward banana only recently. We not only completed the *de novo* assembly of the FS66 genome, which is more superior on contig N50 than the previously available genome of *Fs* str. NRRL 66326 from sugarcane, but also acquired gene structure annotation for the species for the first time. Through comparative genomic analysis among FS66 and closely related Fusarium genomes, lineage-specific characteristics were identified in FS66 including gene gain and loss and gene transfer from other species. The discovery and analysis of this study should have provided important information and clues for further understanding of the molecular mechanism underlying the development of phytopathogenicity in *Fusarium* species.

The BLB disease caused by *Fs* str. FS66, which mostly caused only growth retard of plants in the field, is much less destructive than the deadly Panama disease caused by *Foc* (Dita et al., 2018). *Fs* has been reported to be transmitted by air currents and easily dispersed by rain splash (Nordahliawate et al., 2008), and it has also been reported to cause leaf diseases on *Rhynchostylis gigantea* (Dekham and Kanchanawatee, 2020) and sugarcane (Yao et al., 2020). It has also been reported to cause wilt on sugarcane (Viswanathan et al., 2017; Bao et al., 2020), indicating it has the potential to become a soil-borne disease. A most notorious soil-borne banana pathogen is *Foc*, which first invades the roots and then climbs up the pseudo-stems through the vessels of the plant’s vascular system (Czislowski et al., 2018). Thus in the Panama disease, discolorations of root and pseudo-stem vascular vessels are generally observed, and the leaf wilt symptoms usually first appear on older leaves. No root or pseudo-stem lesion has been observed on banana plants with BLB disease in the field but only on the leaves, and no symptom was observed on plants subjected to root inoculation, showing that FS66 is not a soil-borne pathogen like *Foc*. Another banana disease caused by an FFSC species (*F. verticillioides*) is the pseudo-stem heart rot disease, which also could not invade through the roots and requires inoculation with a wound in the pseudo-stem above the corm for the symptoms to develop (Shalaby et al., 2006). The low successful rate of non-wound inoculation and high successful rate of wound inoculation of FS66 suggests that wounds facilitate or are even necessary in the BLB disease.

Widespread gene transfer signals have been detected in the genome of FS66, suggesting the scale of gene transfer in *Fusarium* genomes could have been underestimated previously. Gene transfer studies in *Fusarium* have mainly been focused on specific genomic regions or a limited number of genes. A review on *Fusarium* pathognomics suggested that the transfer of an entire plasmid or non-core chromosome was the more common type of HGT in *Fusarium* (Ma et al., 2013). Recent studies showed that gene transfer not only occurred on lineage-specific supernumerary chromosomes (Ma et al., 2010; Rocha et al., 2016; van Dam and Rep, 2017; van Dam et al., 2017; Yang et al., 2020) but also on genes located on the core chromosomes (Gardiner et al., 2012; Sieber et al., 2014; Vlaardingerbroek et al., 2016; Hoogendoorn et al., 2018; Liu et al., 2019; Tralamazza et al., 2019) or mitochondrial genomic regions (Brankovics et al., 2020). Most of these studies focused on effectors such as several *SIX* (Secreted In Xylem) genes (van Dam and Rep, 2017; Czislowski et al., 2018) and secondary metabolite gene clusters (Liu et al., 2019; Kim et al., 2020), which are related to the pathogenicity or virulence of *Fusarium* isolates (Lanubile et al., 2016; An et al., 2019). In this study, no homolog of the *Fo* accessory chromosomes was found in FS66, but hundreds of genes were inferred to be putatively transferred from FOSC or other species on the core chromosomes, not only including genes related to pathogenicity or virulence in other *Fusarium* pathogens but also many genes involved in multiple functional categories. The mechanism of gene transfer among *Fusarium* isolates remains largely unknown. Considering *Fusarium* harbors species both with known sexual cycle and those without (Ma et al., 2013), both sexual (introgression) and asexual (HGT) processes should have contributed to the observed gene transfer events.

In conclusion, we reported the BLB disease caused by *Fs* str. FS66 for the first time and obtained both a high-quality assembly and gene structure annotation for the genome. The genome of FS66 shares relatively high nucleotide similarity (96.74%) with *Fs* str. NRRL 66326 from sugarcane, a larger proportion (8.58% of NRRL 66326 and 14.47% of FS66) of the genomes could not be aligned, which could probably be explained by lineage-specific deletion and gene transfer events. Hundreds of genes across the FS66 genome putatively transferred from FOSC and other outgroup species were detected in the FS66 genome, including a few involved in pathogenicity/virulence. These results and analyses should be valuable for further understanding of the genomic evolution underlying new pathogenicity development in *Fusarium*.

## Data Availability Statement

The genome sequence ( doi: 10.6084/m9.figshare.13524434) and gene structure annotations ( doi: 10.6084/m9.figshare.13524443) have been deposited to both NCBI (BioProject PRJNA667564) and the FigShare repository. The DDBJ/ENA/GenBank accession ID of the FS66 genome assembly is JADANP000000000, and the version described in this paper is version JADANP010000000. Raw sequencing data of genome and transcriptome have been deposited under the same NCBI BioProject. Whole-genome mRNA sequences ( doi: 10.6084/m9.figshare.13524452), coding sequences ( doi: 10.6084/m9.figshare.13524458), protein sequences ( doi: 10.6084/m9.figshare.13524449), gene function annotation ( doi: 10.6084/m9.figshare.13524446), repeat element annotation ( doi: 10.6084/m9.figshare.13524497), non-coding RNA annotation ( doi: 10.6084/m9.figshare.13524464), and mitochondria genome annotation ( doi: 10.6084/m9.figshare.13524467) files are accessible from FigShare.

## Author Contributions

YC and BW conducted data analysis and manuscript writing. YC, AP, and XS carried out field investigation. YC carried out all the experiments. All authors participated in the revision of the manuscript. All authors have read and agreed to the published version of the manuscript.

## Conflict of Interest

The authors declare that the research was conducted in the absence of any commercial or financial relationships that could be construed as a potential conflict of interest.

## Footnotes

1 https://github.com/TransDecoder/TransDecoder

2 https://github.com/tseemann/barrnap

3 http://mitos2.bioinf.uni-leipzig.de/index.py
