# Supplementary material for: The Genome of Banana Leaf Blight Pathogen Fusarium sacchari str. FS66 Harbors Widespread Gene Transfer From Fusarium oxysporum
Source: Front Plant Sci. 2021 Feb 4;12:629859. doi: 10.3389/fpls.2021.629859 (PMC7889605; doi:10.3389/fpls.2021.629859)
Supplement: Supplementary file 2 [file Data_Sheet_1.docx]

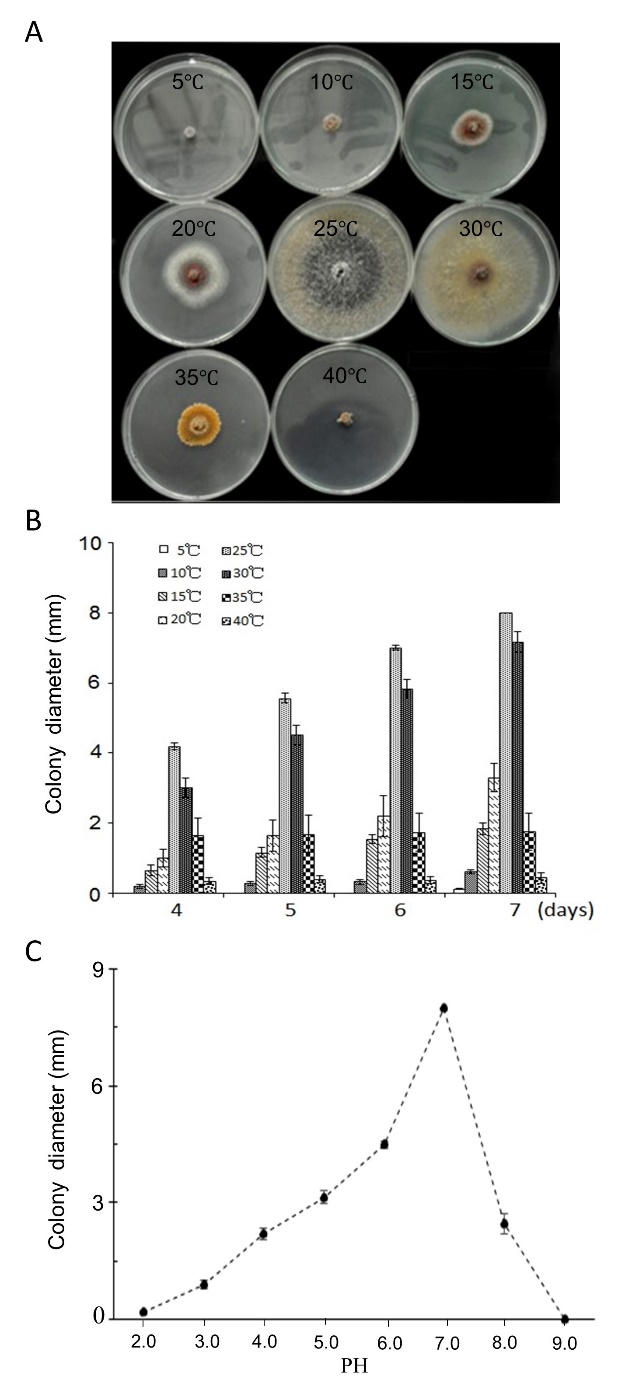


**Supplemental Figure 1.** The growth of FS66 colonies under different conditions. All the error bars denote standard error calculated based on three replicates of experiments. (A and B) FS66 colonies growing on PDA plates (pH = 7.0) under different temperatures at 7 days (A) after single spore inoculation and their diameters from day 4 to day 7 (B). (C) Diameters of FS66 colonies growing on different PDA plates with adjusted pH values at day 7 after inoculation.


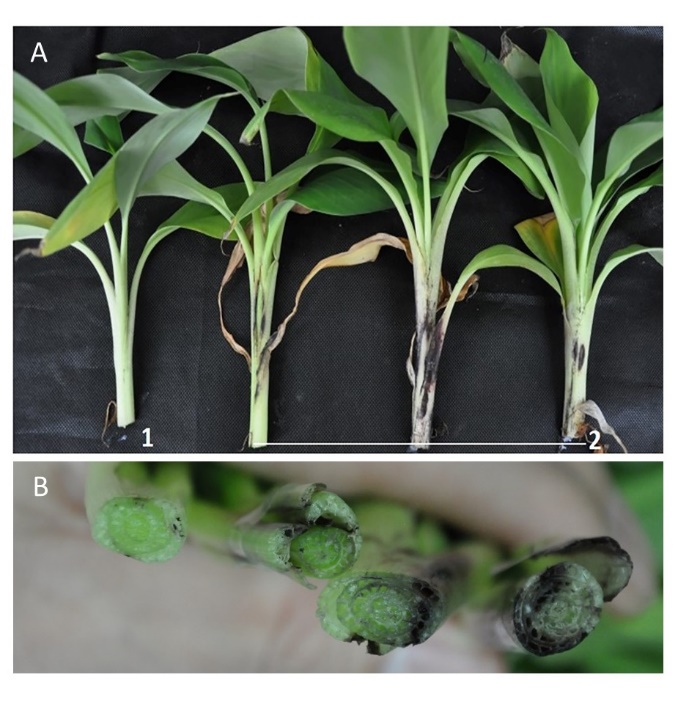


**Supplemental Figure 2.** Symptom development on banana pseudo-stems inoculated with FS66. (A) Symptoms of seedlings at 45 days after inoculation with sterile water (1) and FS66 (2) on the pseudo-stems punctured by needles. (B) The pseudo-stem crossing sections of the four seedlings in Figure A shown in the same order.


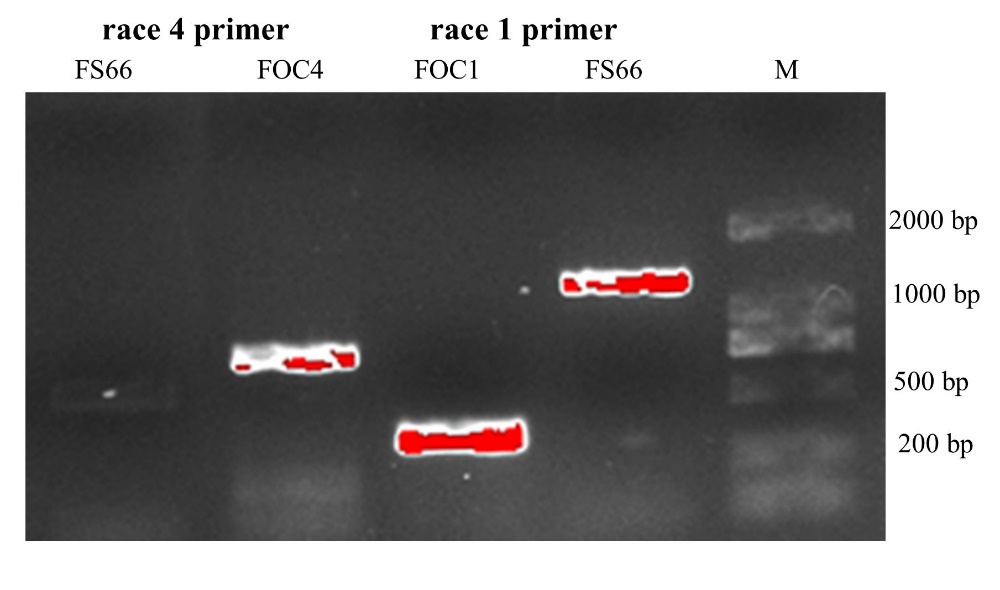


**Supplemental Figure 3. PCR amplification of FS66 template with *Foc* race 1 and race 4 specific primers.** FOC1 and FOC4 were *Foc* race 1 and tropical race 4 isolates previously collected by our lab, respectively.


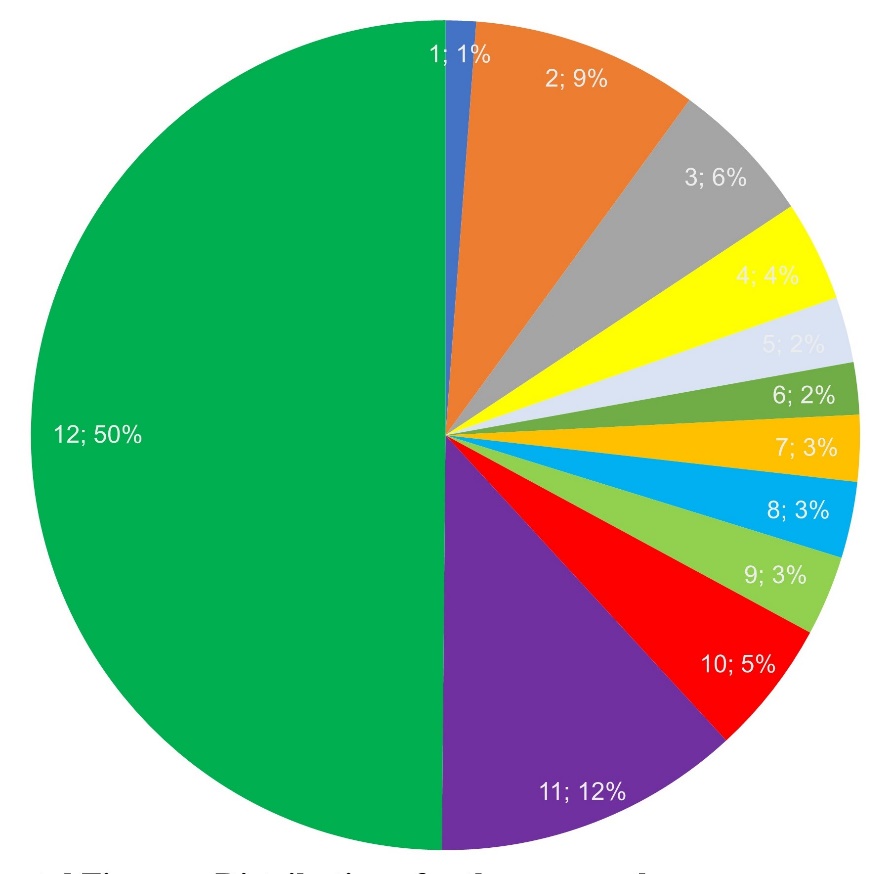


**Supplemental Figure 4. Statistics on the number of genomes present in the orthogroups.** An integer and a percentage value have been shown in each label. The integer denotes the number of genomes which harbor at least one member in the orthogroups, and the percentage denotes the proportion of orthogroups.
